# Perioperative antibiotics in pediatric cardiac surgery: protocol for a systematic review

**DOI:** 10.1186/s13643-017-0502-y

**Published:** 2017-05-30

**Authors:** Vijay Anand, Angela Bates, Robin Featherstone, Srinivas Murthy

**Affiliations:** 1 University of Alberta, Edmonton, Canada; 2 University of Pennsylvania, Philadelphia, USA; 3 University of British Columbia, 4500 Oak Street, Vancouver, Canada

**Keywords:** Healthcare-associated infections, Pediatrics, Cardiac surgery

## Abstract

**Background:**

Post-operative infections in pediatric cardiac surgery are an ongoing clinical challenge, with rates between 1 and 20%. Perioperative antibiotics remain the standard for prevention of surgical-site infections, but the type of antibiotic and duration of administration remain poorly defined. Current levels of practice variation through informal surveys are very high. Rates of antibiotic-resistant organisms are increasing steadily around the world.

**Methods/design:**

We will identify all controlled observational studies and randomized controlled trials examining prophylactic antibiotic use in pediatric cardiac surgery. Data sources will include MEDLINE, EMBASE, CENTRAL, and proceedings from recent relevant scientific meetings. For each included study, we will conduct duplicate independent data extraction, risk of bias assessment, and evaluation of quality of evidence using the GRADE approach.

**Discussion:**

We will report the results of this review in agreement with the PRISMA statement and disseminate our findings at relevant critical care and cardiology conferences and through publication in peer-reviewed journals. We will use this systematic review to inform clinical guidelines, which will be disseminated in a separate stand-alone publication.

**Study registration number:**

PROSPERO CRD42016052978C

**Electronic supplementary material:**

The online version of this article (doi:10.1186/s13643-017-0502-y) contains supplementary material, which is available to authorized users.

## Background

### Description of the problem

Post-operative infections in pediatric cardiac surgery remain an ongoing clinical challenge. The burden of disease has a wide range, dependent on the case series examined, ranging from approximately 1 to 18% in children with delayed sternal closure [1–4]. There are many factors that contribute to increased risk of infection, including overall acuity, age, delayed sternal closure, steroid use, and length-of-stay in ICU [5–8]. The presence of infection is associated with worsened outcomes and increased costs [9].

Antibiotic use in the perioperative period are well-established adjuncts to reducing the incidence of infection [10]; however, the nature, timing, and duration of administration remain undetermined. Further, in the context of increasing attention to antimicrobial resistance predicated upon the overuse of antibiotics, addressing this issue is timely [11].

### Description of the intervention

Antibiotic prophylaxis for surgical procedures is a well-established practice that reduces surgical-site infections. By preventing translocation of bacteria from the skin, antibiotic prophylaxis reduces the rate of post-operative infections in all types of procedures, from clean to dirty. As a clean procedure, pediatric cardiac surgeries should have a lower risk for infection; however, given the severity of illness and prolonged stays in intensive care, infections remain an ongoing challenge.

Antibiotic prophylaxis in pediatric cardiac surgery takes numerous forms. Regimens vary greatly, from single-dose prophylaxis to continuing antibiotics until all chest tubes and central venous catheters have been removed [10]. In children with delayed sternal closure, antibiotic regimens vary again, from 48 h of antibiotics to antibiotics continuing until chest closure has been achieved. Further, the type of antibiotic used varies; although, this is primarily contingent upon the endemic organisms present in specific institutions, i.e., vancomycin for high rates of MRSA.

### Why is it important to conduct this review

Given the issue of antimicrobial resistance and a focus on antimicrobial stewardship in critical care, the varied rates of post-operative infections, and the incredibly diverse regimens used for antibiotic prophylaxis, it is timely to systematically review the literature to determine the optimal strategy to prevent infections in critically ill children. Further, data guidance from adult-specific randomized trials are less relevant to children, given the very different physiology and infectious risks in the two cohorts [12, 13].

### Research question

Is a shortened course of perioperative antibiotics in children undergoing cardiac surgery as safe as a prolonged course of perioperative antibiotics?

## Methods and analysis

### Criteria for selecting studies

#### Types of studies

We will include all controlled observational studies (case-control or cohort) and randomized trials, excluding case reports or case series, with no restrictions based on language or quality. We will only include papers published after 1990, given the large changes in practice since that point in time in pediatric cardiac surgery.

#### Types of participants

The population of interest is children (<18 years) undergoing open heart surgery.

#### Types of interventions

The interventions examined include any systemic antibiotic regimen used for the prevention of infection in children having undergone cardiac surgery. We will include studies that report the nature (drug, duration) of antibiotics administered. We will exclude studies that exclusively report antibiotics used for the treatment of established infections. We will exclude studies that exclusively report on the pre-operative use of decolonization regimens.

#### Types of outcome measures

We will include studies that report the incidence of infection, as defined by the individual studies. Other outcomes of interest include, if reported: length-of-ICU-stay, mortality, cost of care, antibiotic-associated adverse events, and presence of antibiotic-resistant organisms (as defined by individual authors).

### Search methods

We will perform a search of the following databases for relevant studies: MEDLINE, EMBASE, and the Cochrane Central Register of Controlled Trials (CENTRAL). The peer-reviewed MEDLINE search strategy is included in Additional file 1, with similar searches with adapted keywords for other databases. To locate in-process and unpublished studies, we will also search trial registries, ClinicalTrials.gov and the World Health Organization’s International Clinical Trial Registry Platform (WHO ICTRP), from 2014 to 2017.

We will screen reference lists of included studies and relevant reviews for eligible articles. We will also manually screen conference proceedings from 2014 to 2017 for the following scientific meetings: Society of Critical Care Medicine, Pediatric Cardiac Intensive Care Society, Society of Thoracic Surgeons, American Heart Association, and World Congress of Pediatric Cardiology and Cardiac Surgery.

Search results will be exported to the EndNote X7 citation manager program. Preliminary scoping searches have been performed and no randomized trials have been found.

### Study records

Pairs of two reviewers will independently screen titles and abstracts using a pretested electronic screening form (www.covidence.org), including any article for full-text review unless both reviewers exclude. Pairs of two reviewers will then independently screen all full-text articles using specific eligibility criteria through this platform, resolving disagreements by consensus, and reporting a Cohen’s k for full-text eligibility screening.

### Data collection

Teams of two reviewers will perform data extraction independently and in duplicate using data collection forms through Covidence, collecting information pertaining to the study design, patient characteristics, intervention (and comparator, if applicable), and clinical outcomes. Interventions will include specifying the antibiotic used, doses (if available), the duration of use, and specific reasons for altering these practices. Outcomes will include a primary outcome of incidence of nosocomial infection (as defined by the individual paper), mortality, duration of mechanical ventilation, and duration of intensive care unit stay. Conflicts will be resolved through discussion.

### Risk of bias assessment

For observational studies, we will use the risk of bias tools for cohort and case-control studies developed by the Clinical Advances Through Research and Information Translation (CLARITY) group at McMaster university [14, 15]. These tools evaluate the selection of groups, the adequacy of assessment of prognostic factors, the assessment of exposures and outcomes, and the similarity of co-interventions between groups. We will assess the overall quality of data for our primary outcome using the GRADE approach.

### Summarizing data and treatment effect

Given the expected heterogeneity of the study designs, we will not perform a meta-analysis. We will provide quantitative summaries where available of relevant treatment effects of different antibiotic treatment durations of individual studies, with tabular results of included studies.

### Subgroup analysis and investigation of heterogeneity

Subgroup overviews will be performed for children with delayed sternal closure. Given the absence of a planned meta-analysis, quantitative subgroup analysis will be deferred.

## Discussion

Perioperative infections are a common cause of post-operative morbidity in this high-risk population. Optimizing the antibiotic regimens for these children is a frequent clinical conversation that is woefully understudied despite its widespread practice. As a comparative effectiveness program, we aim to determine the best method to prevent infections, without increasing the adverse effects of antibiotics such as increasing resistance and individual adverse effects.

### Ethics and dissemination

We did not require ethics approval for this study. We will report this review in accordance with the PRISMA statement [16]. This protocol has been registered at the PROSPERO database (CRD42016052978C) and is reported in accordance with the Preferred Reporting Items for Systematic Reviews and Meta-Analyses Protocols (PRISMA-P) guidelines (see Additional file 2) [17]. There is no specific funding attached to this systematic review. We will disseminate our findings by producing clinical guidelines, as well as conference presentations and publication in a peer-reviewed journal.

## Additional files

Additional file 1: Search Strategy, filename: Appendix 1. (DOCX 84 kb)

Additional file 2: PRISMA-P Checklist, filename: PRISMA-P 2015 checklist antibiotics. (DOCX 37 kb)

## Abbreviations

CLARITY: Clinical advances through research and information translation
PRISMA: Preferred Reporting Items for Systematic Reviews and Meta-Analyses

## References

[1] Tortoriello TA, Friedman JD, McKenzie ED (2003). Mediastinitis after pediatric cardiac surgery: a 15-year experience at a single institution. Ann Thorac Surg.

[2] Al-Sehly AA, Robinson JL, Lee BE (2005). Pediatric poststernotomy mediastinitis. Ann Thorac Surg.

[3] Nelson-McMillan K, Hornik CP, He X (2016). Delayed sternal closure in infant heart surgery-the importance of where and when: an analysis of the STS congenital heart surgery database. ann thorac surg.

[4] Agus MS, Steil GM, Wypij D (2012). Tight glycemic control versus standard care after pediatric cardiac surgery. N Engl J Med.

[5] Bowman ME, Rebeyka IM, Ross DB, Quinonez LG, Forgie SE (2013). Risk factors for surgical site infection after delayed sternal closure. Am J Infect Control.

[6] Mastropietro CW, Barrett R, Davalos MC (2013). Cumulative corticosteroid exposure and infection risk after complex pediatric cardiac surgery. Ann Thorac Surg.

[7] Ben-Ami E, Levy I, Katz J, Dagan O, Shalit I (2008). Risk factors for sternal wound infection in children undergoing cardiac surgery: a case-control study. J Hosp Infect.

[8] Kagen J, Lautenbach E, Bilker WB (2007). Risk factors for mediastinitis following median sternotomy in children. Pediatr Infect Dis J.

[9] Braxton JH, Marrin CA, McGrath PD (2004). 10-year follow-up of patients with and without mediastinitis. Semin Thorac Cardiovasc Surg.

[10] Alphonso N, Anagnostopoulos PV, Scarpace S (2007). Perioperative antibiotic prophylaxis in paediatric cardiac surgery. Cardiol Young.

[11] Holmes AH, Moore LS, Sundsfjord A (2016). Understanding the mechanisms and drivers of antimicrobial resistance. Lancet.

[12] Harbarth S, Samore MH, Lichtenberg D, Carmeli Y (2000). Prolonged antibiotic prophylaxis after cardiovascular surgery and its effect on surgical site infections and antimicrobial resistance. Circulation.

[13] Bratzler DW, Dellinger EP, Olsen KM (2013). Clinical practice guidelines for antimicrobial prophylaxis in surgery. Am J Health Syst Pharm.

[14] Tool to Assess Risk of Bias in Case-control Studies. (Accessed 24 Apr 2017 at https://www.evidencepartners.com/wp-content/uploads/2014/02/Tool-to-Assess-Risk-of-Bias-in-Case-Control-Studies-Aug-21_2011.doc).

[15] Tool to Assess Risk of Bias in Cohort Studies. (Accessed 24 Apr 2017, at https://www.evidencepartners.com/wp-content/uploads/2014/02/Tool-to-Assess-Risk-of-Bias-in-Cohort-Studies.doc).

[16] Moher D, Liberati A, Tetzlaff J, Altman DG, Group P (2009). Preferred reporting items for systematic reviews and meta-analyses: the PRISMA statement. PLoS Med.

[17] Moher D, Shamseer L, Clarke M (2015). Preferred Reporting Items for Systematic Review and Meta-Analysis Protocols (PRISMA-P) 2015 statement. Syst Rev.

