# Supplementary material for: Perioperative antibiotics in pediatric cardiac surgery: protocol for a systematic review
Source: Syst Rev. 2017 May 30;6:107. doi: 10.1186/s13643-017-0502-y (PMC5450342; doi:10.1186/s13643-017-0502-y)
Supplement: Supplementary file 1 — Search Strategy, filename: Appendix 1. (DOCX 84 kb) [file 13643_2017_502_MOESM1_ESM.docx]

**Appendix 1**

**Ovid Medline Search strategy**

1. Ampicillin/

2. Anti-Bacterial Agents/

3. Antibiotic Prophylaxis/

4. Bacterial Infections/pc

5. Cefazolin/

6. Cefoxitin/

7. Cephalosporins/

8. Clindamycin/

9. Cross Infection/pc

10. Fluoroquinolones/

11. Gentamicins/

12. Mediastinitis/pc

13. Premedication/

14. Staphylococcal Infections/pc

15. Surgical Wound Infection/pc

16. Vancomycin/

17. amp#c#llin*.tw,nm,kf.

18. (anti bacter* or antibacter*).tw,kf.

19. (anti bioprophyla* or antibioprophyla*).tw,kf.

20. (anti biotic* or antibiotic*).tw,kf.

21. (anti microb* or antimicrob*).tw,kf.

22. (cefazolin* or cephazolin*).tw,nm,kf.

23. (cefepime* or cephepime*).tw,nm,kf.

24. (cefoxitin* or cephoxitin*).tw,nm,kf.

25. (cefuroxime* or cephuroxime*).tw,nm,kf.

26. clindam#cin*.tw,nm,kf.

27. fluoroquinolone*.tw,nm,kf.

28. gentam#cin*.tw,nm,kf.

29. mediastinitis*.tw,kf.

30. metronidazole*.tw,nm,kf.

31. vancom#cin*.tw,nm,kf.

32. or/1-31 [Combined MeSH & text words for antibiotics]

33. Anastomosis, Surgical/ and (cardi* or coronary or heart* or pulmon* or thoracic or thorax).mp.

34. exp Cardiac Surgical Procedures/

35. exp Heart/su

36. exp Heart Defects, Congenital/su

37. exp Heart Transplantation/

38. Sternotomy/

39. Sternum/su

40. Thoracic Surgery/

41. ((AAOCA or aort* or IAA) adj3 (repair* or reconstruct*)).tw,kf.

42. ((AICD* or ICD* or implantable cardioverter-defibrillator) adj3 (implant* or procedur*)).tw,kf.

43. aortopex*.tw,kf.

44. ((arterial or atrial) adj2 switch*).tw,kf.

45. ((ASD or atrial septal defect* or ventricular septal defect* or VSD) adj3 (reconstruct* or repair*)).tw,kf.

46. atrial baffle*.tw,kf.

47. ((atrioventricular septal defect* or AVC or AVSD* or PAPCV or PAPVC of PAVSD) adj3 (reconstruct* or repair*)).tw,kf.

48. (band* adj3 (LPA or PA or pulmonary arter* or RPA)).tw,kf.

49. (BBDCPA or ((bi-directional or bidirectional) adj2 cavopulmonary anastomosis) or ((bi-directional or bidirectional) adj2 Glenn)).tw,kf.

50. ((cardi* or coronary or heart*) adj3 (angioplast* or atherectom* or by-pass* or bypass* or graft* or implant* or perioperativ* or peroperativ* or postoperativ* or procedur* or operat* or repair* or reconstruct* or resect* or surg* or transplant*)).tw,kf.

51. Blalock-Taussig*.tw,kf.

52. chest closure*.tw,kf.

53. (clos* adj3 (sternal* or sternum*)).tw,kf.

54. (coarctation adj3 repair*).tw,kf.

55. (congenital heart defect* and (perioperative or peroperativ* or postoperativ* or procedur* or operat* or repair* or reconstruct* or surg* or transplant*)).tw,kf.

56. (Damus-Kaye-Stansel or (DKS adj2 procedur*)).tw,kf.

57. double switch*.tw,kf.

58. (Fontan adj2 procedur*).tw,kf.

59. Glenn shunt*.tw,kf.

60. (mitral valve adj3 (reconstruct* or repair*)).tw,kf.

61. (mitral valve replace* or (MVR adj3 (procedur* or surg*))).tw,kf.

62. ((myocardi* or transmyocardi*) adj2 revascular*).tw,kf.

63. Norwood*.tw,kf.

64. ((PA or pulmonary atresia*) adj3 (band* or deband* or reconstruct* or repair*)).tw,kf.

65. ((pace maker* or pacemaker*) adj3 (implant* or procedur*)).tw,kf.

66. ((patent ductus arteriosus or PDA) adj3 closure*).tw,kf.

67. ((patent foramen ovale or PFO) adj3 closure*).tw,kf.

68. (peri-cardiectom* or pericardiectom*).tw,kf.

69. (poststernotom* or sternotom*).tw,kf.

70. (pulmon* arter* adj3 (reconstruct* or repair*)).tw,kf.

71. pulmon* embolectom*.tw,kf.

72. (pulmon* ven* adj3 (reconstruct* or repair*)).tw,kf.

73. Rastelli*.tw,kf.

74. ((right ventricular outflow tract or RVOT) adj3 (implant* or procedur* or reconstruct*)).tw,kf.

75. (Ross adj2 procedur*).tw,kf.

76. sternal wound infection*.tw,kf.

77. (surgical procedure* and (neonatal intensive care or NICU* or p?ediatric intensive care or PICU*)).tw,kf.

78. (TAPVC* or total anomalous pulmonary venous connection).tw,kf.

79. ((transposition of the great arteries or TGA) adj2 repair*).tw,kf.

80. (truncus adj2 repair*).tw,kf.

81. (valve adj3 (annuloplast* or excis* or repair* or replace*)).tw,kf.

82. valvotom*.tw,kf.

83. valvuloplast*.tw,kf.

84. ((ventricular septal defect* or VSD*) adj3 (reconstruct* or repair*)).tw,kf.

85. ventriculectom*.tw,kf.

86. or/33-85 [Combined MeSH & text words for cardiac surgery]

87. and/32,86 [Combined Concepts for antibiotics & cardiac surgery]

88. Adolescent/

89. exp Child/

90. exp Infant/

91. Minors/

92. exp Pediatrics/

93. (baby* or babies or infant* or infancy or neo-nat* or neonat* or newborn*).tw,jw,kf.

94. (boy* or girl* or minors or teen*).tw,kf.

95. (child* or kid or kids or preschool* or school age* or schoolchild* or toddler*).tw,jw,kf.

96. (elementary school* or high school* or highschool* or kindergar* or nursery school* or primary school* or secondary school*).tw,kf.

97. p?ediatric*.tw,kf,jw.

98. or/88-97 [Combined MeSH & text words for children]

99. and/87,98 [Child filter applied to combined concepts for antibiotics & cardiac surgery]

100. exp Animals/ not Humans/

101. (animal or animals or canine* or cat or cats or dog or dogs or feline or felines or hamster or hamsters or mice or monkey or monkeys or mouse or pig or piglet or piglets or pigs or porcine or primate* or rabbit or rabbits or rat or rats or rodent or rodents or sheep or swine or swines).ti.

102. 99 not (100 or 101) [Animal studies filter applied to combined concepts for antibiotics & cardiac surgery with child filter]

103. limit 102 to yr="1990-Current"

104. remove duplicates from 103
